# Patterns and epidemiology of acute poisoning in Ethiopia: systematic review of observational studies

**DOI:** 10.1186/s13690-018-0275-3

**Published:** 2018-07-02

**Authors:** Legese Chelkeba, Abera Mulatu, Dessalegn Feyissa, Firomsa Bekele, Behailu Terefe Tesfaye

**Affiliations:** 1 School of pharmacy, Department of Clinical pharmacy, College of Health Sciences, Jimma University, Jimma, Ethiopia; 2 Amanuel Mental Specialized Hospital, Addis Ababa, Ethiopia; 3 Mizan Tepi University School of pharmacy, Clinical Pharmacy Unit, Mizan, Ethiopia; 4 Metu UniversitySchool of pharmacy, Clinical Pharmacy Unit, Metu, Ethiopia

**Keywords:** Epidemiology, Pattern, Poisoning, Ethiopia

## Abstract

**Background:**

Acute poisoning is a common reason for emergency department visit and hospitalization worldwide with major morbidity and mortality. The burden of poisoning exposures in Africa is a significant public health concern, but only 10 of 58 countries have poisons information centers (PICs).

**Objective:**

The primary intention of our current review is to explore and summarize the published evidence on the patterns and epidemiology of poisoning in Ethiopia.

**Method:**

PubMed and Scopus were searched for primary, case series and human studies for publications from inception to July 2017. A manual search for additional relevant studies using references from retrieved articles was also performed. Only studies that reported acute poisoning in both pediatric and adult patients were included. From the screened articles, data were extracted for baseline characteristics and relevant end points such as case fatality rate, time for health institution presentation and length of hospital stay.

**Result:**

Initial entry and search resulted in the retrieval of 332 articles. Finally, 9 studies comprised of 4763 participants were included in this current review. In 78% of the studies included in this review, acute poisoning is reported to be more prevalent in females. Acute poisoning was revealed to be prevalent in less than 30 years old. Organophosphates and household cleaning agents were the predominant agents of acute poisoning. Intentional poisoning was identified responsible for the majority of acute poisoning cases and factors such as psychiatric problems, and quarrel were identified as the underlying reasons for poisoning. Time of presentation to health institution after poisoning, length of hospital stay and case fatality rate were reported and lies in the ranges between 0.2 h–24 h, 0.5 days–17.7 days and 0–14.8%, respectively.

**Conclusion:**

The occurrence of acute poisoning was higher in females and common in less than 30 years of age, making this a real public health burden in Ethiopia. Psychiatric problems, quarrel and substance abuse were identified as the most common reasons for acute poisoning. Awareness creation how to handle chemicals and prescribed drugs and psychiatric consultations should be in place for the community.

## Introduction

Poison is a substance that causes damage or injury to the body and endangers one’s life [1]. Normally used substances such as water can be poisonous depending on the amount ingested [2]. Chronic poisonings or poisonings with delayed health effects are often more problematic in the long run [3]. If the toxic effects occur immediately, usually within hours from the time of exposure, it is called acute poisoning [4]. Poisoning is a common reason for emergency departments visit and hospitalization worldwide with major morbidity and mortality in many countries [4]. It is the third most common emergencies of pediatrics leading to increased childhood morbidity and mortality [5]. It could be intentional or unintentional, and because of their exploratory nature and their desires to imitate adults, the unintentional or accidental poisoning is common among children [6].

Poisoning is a significant global public health problem and cases are increasing day to day due to changes in the life style and social behavior [7]. Advances in technology and social development have resulted in the availability of most drugs and chemical substances in the community. The exact number of incidences can be higher, because most cases of poisoning actually go unreported [8]. World health organization (WHO) data of 2012 shown that, worldwide, approximately 193,460 people were died due unintentional poisoning, 84% of which occurred in low- and middle-income countries. Loss of over 10.7 million years of healthy life (disability adjusted life years, DALYs) were also observed in the same year [7].

There are many differences with respect to the pattern and cause of acute poisoning between geographical regions, even within the same country. The knowledge of the general pattern of poisoning in a particular region would help to identify the risk factors and allow early diagnosis and management of such cases, which in turn should result in reduction of morbidity and mortality [8]. For example, high doses of analgesics, tranquillizers, and antidepressants are the commonly used agents for intentional poisoning in industrialized countries and agriculture pesticides are used in Asian region for self-poisoning particularly in rural areas with a fatality range of 10–20%. Majority of pesticide exposure is seen more in middle and low income countries due to increased use of agrochemicals in agricultural sector [1].

Animal envenomations are a problem in many areas of the world that are home for poisonous snakes, spiders and scorpions [6]. It has been estimated that about 5 million snake-bites occur each year, resulting in up to 2.5 million envenomings, at least 100,000 deaths and around three times as many amputations and other permanent disabilities [7]. In terms of DALYs, the impact of envenomings is very high and estimated in 2 million DALYs per year for sub-Saharan Africa, because most victims are children or young agricultural workers, many of whom are left for the rest of their lives with permanent physical or psychological consequences of envenoming [9].

## Poisoning in Africa

Significant morbidity and mortality is associated with acute pesticide poisoning, especially in developing countries. In these countries no reliable data is available as to how many people per year suffer from pesticide-related health effects. For this several reasons have been hypothesized including a lack of standardized case definition [10–12]. As part of the developing world, even though the burden of poisoning exposures in Africa is a significant public health concern, only ten of 58 countries (17.2%) have poisons information centers (PICs). In this region, since poisoning cases are usually poorly documented, the genuine epidemiology and accurate figures of acute poisoning is unknown. The underlying reasons are lack of resources and knowledge to diagnose poisoning, the fact that only certain acute poisoning cases are required to be reported to the local or national department of health, and low levels of death. Based on data from 2012, WHO estimated that unintentional poisoning accounts for 39,800 deaths and 27,949,000 DALYs in the Africa region [13].

## Poisoning in Ethiopia

In Ethiopia, epidemiological data on acute poisoning is extremely few and it is very difficult to find primary data. The culprits of this problem are unavailability of well-organized poison control center and routine screening & confirmatory tests. Few published epidemiological studies with small sample size exist concerning acute poisoning. To our best knowledge, this is the first systematic review that summarized comprehensive data on acute poisoning in the country.

## Objective

The objective of this review is to explore and summarize the published evidence on the patterns and epidemiology of acute poisoning in Ethiopia.

## Methods

### Data sources and searching procedures

Four independent reviewers (AM, BT) were searching for relevant literatures in PubMed and Scopus databases and the third author (LCH) was consulted for disagreement of the relevance of the studies to be included in the review. A search was conducted for primary articles, case series and human studies published in English language without limiting year of publication not to miss important literatures. A search was conducted on July 2017 using the search terms “patterns” OR “incidence” OR “prevalence” OR “epidemiology” AND “poisoning” OR “toxicity” OR “intoxication” AND “Ethiopia”. A manual search for additional relevant studies using references from retrieved articles was also performed. The search strategy and search terms used in this study are detailed in (Appendix). The full in the initial screening stage, two investigators independently reviewed the title and abstract of each the references to exclude articles irrelevant to the systematic review using rigorous inclusion criteria. In the second stage, the two investigators independently read the full texts of the articles that were not excluded in the initial stage, and then selected the articles that met the inclusion criteria. Differences of ideas regarding the selection of articles were resolved through discussion.

### Inclusion and exclusion criteria

Only primary articles and case series of acute poisoning of human studies published in English language, which contain relevant end points are included. Both pediatric and adult studies which met the preceding criteria are eligible. Studies were excluded from our review if any one or more of the following conditions applied: chronic poisoning studies, the outcomes of interest were not clearly reported, unpublished studies, non-English and/or non-human studies.

### Data abstraction

The authors extracted data from eligible studies onto a standardized data abstraction sheet. We extracted information on name of first author and year of publication, study designs, average age (years), gender, residence, marital status, religion, study setting, responsible agents for acute poisoning, route of poisoning, number of participants, circumstances(intentional, unintentional poisoning or undetermined), clinical presentation, pre- and hospital treatment, reason (s) for poisoning and patient outcome.

#### Methodology quality assessment

The assessment of methodological quality was carried out independently by two reviewers using the Newcastle-Ottawa scale (NOS). A quality scale was calculated based on three categories.

1. Selection (maximum 4 stars)
2. Comparability between groups (maximum 2 starts)
3. Outcome assessment (maximum 3 stars). A maximum of one star could be awarded for each item in the group selection and outcome assessment categories. A maximum of two stars could be awarded for comparability. Therefore, the maximum possible score was 9 stars, which represented the highest methodological quality. Studies were labeled as having high quality if the score was above the average (8 stars). Disagreements between the reviewers were resolved through discussion.

## Result

**Fig. 1:**
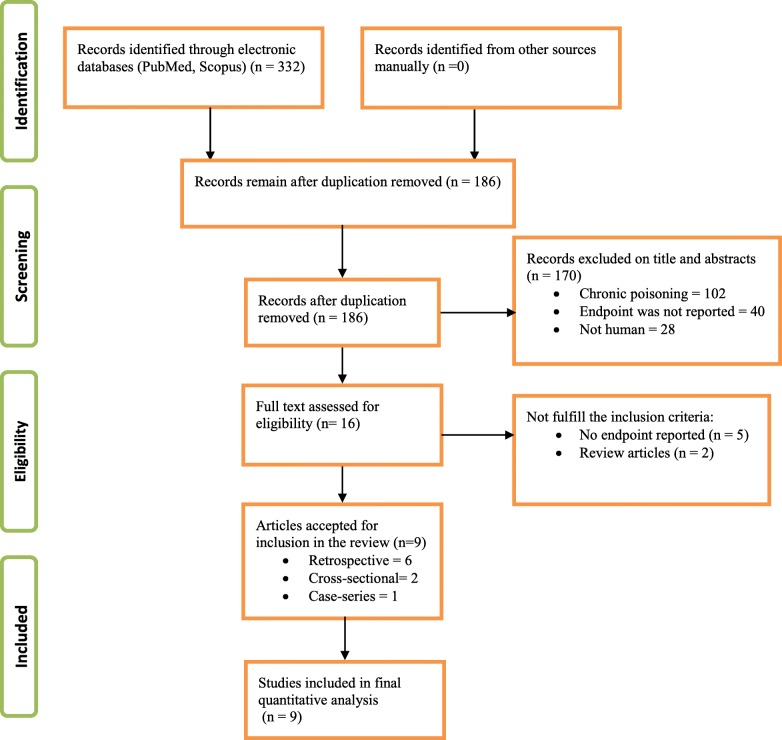
Flowchart for selection of studies

Initially, a total of 332 articles were retrieved from two databases (PubMed and Scopus) and other relevant literatures. Limiting search for primary articles, case series and human studies published in the English language and removal of duplicates and irrelevant articles resulted in a retrieval of 16 articles. Full texts of these 16 articles were accessed and 9 articles were accepted and considered for final review as shown in Fig. 1.

### Characteristics of studies included in this review

**Studies design**: a total of 9 studies were included in this review, 6 of which retrospective, 2 cross-sectional and 1 case series.

**Studies site descriptions:** studies included in this review were conducted in six different well known referral teaching university hospitals in Ethiopia. Gondar university hospital is located in the Northern West part of Ethiopia and it is a 300- bed referral hospital [14]. Tikur Anbessa Specialized Hospital (TASH) is the largest referral teaching hospital found at the capital city of Ethiopia, Addis Ababa. It has over 700 beds, and serves as a medical and other health sciences training center for undergraduate and postgraduate students [15, 16]. Adama Hospital Medical College (AHMC) is located in Adama town, 98 km east of the capital city of Ethiopia, Addis Ababa. It has a total of 220 beds. In emergency department (ED), the patients are evaluated on arrival and after stabilization they are referred to the appropriate department [2].

**Studies period**: articles included in this review are conducted for a median period of 35.5 months (minimum for 12 months and maximum 192 months). The overall summaries of the studies included in this review are depicted in Table 1.

**Table 1:** Baseline characterstics of victims of acute poisoning as stated by studies

Studies	Study design	Average Age(yr)	Gender (%)		Residence (%)		Marital status (%)				Religion
			Male	Female	Urban	Rural	Single	Married	Divorced	Widowed	
Bacha 2015 [17]	Cross-sectional	5.4 years	47.7	51.6							
Teklemariam 2016 [18]	Retrospective		47.6	52.4			18.45	43.7	26.2	11.7	Muslim (47.6%) Orthodox (35.9%) Protestants (10.7%) Others (5.8%)
Adinew 2017 [14]	Retrospective	25.5 years	40	60	56.7	43.3					
Jemal 2016 [19]	Cross-sectional	25.18 years	47	53	59.7	40.3					Orthodox (51.8%), Muslims (27.4%) Protestants (8.1%)
Adinew 2016 [20]	Retrospective	24.36 years	36.5	63.5	75.97	24.03					
Desalew 2011 [15]	Retrospective	21 years	35.3	64.7	89.6		72.2	22.7	1	1	
Chala 2015 [21]	Retrospective	23.1 years	49.7	50.3	31.2	68.8	55.5	39.7	4.8		
Mekonnen 2016 [22]	Case series	25 years	24	3		88.9					
Melaku 2006 [36]	Retrospective	37.10 years									

### Demographic distribution of poisoning among victims of acute poisoning

The highest prevalence of poisoning was observed in persons under age of 30 years. 78% of studies included in this review revealed that occurrence of acute poisoning are higher in females than males [14, 15, 17–21]. Only one study conducted on snake bite poisoning in 27 adults shown male to female ratio of 8:1 [22] as shown in Table 1. Occupation of the victims of acute poisoning was reported in only two studies; farmers (18.8%), unemployed individuals (18.2%), students (54.3%) and daily laborers (13%) were identified as the predominant victims. Other groups were house wives, daily laborers, waitress, government employee, merchant, and prisoner [15, 21]. Education level of victims of acute poisoning was reported in one study and they were Illiterate (59.2%), primary school (21.4%), secondary school (16.5%), and higher institute (2.9%) level personnel [18]. Reported in one study, places of poisoning were identified to be outside of home 8 (6.3%), Unknown25 (19.5%) [17]. Two studies reported that spring (32.2%) and July (14.6%) were the predominant season and month, respectively, associated with higher occurrence of acute poisoning [1, 20].

### Agents responsible for poisoning

Organophosphates and household cleansing agents were the predominant agents of acute poisoning identified. Other agents stated as cause of acute poisoning in the included studies were pharmaceuticals, hydrocarbons, alcohols, snake bites and so on [14, 15, 17–21].

### Circumstances of poisoning

In our current review, intentional poisoning was reported responsible for majority of acute poisonings [14, 15, 18, 19, 21].

### Reasons for poisoning

Psychiatry problems, quarrel (family, marital, social), family problems, emotional disturbance, and substance abuse (such as alcohol) were identified as underlying reasons for poisoning [14, 15, 18, 21].

### Route of poisoning and signs and symptoms of acute poisoning

Oral ingestion was identified as the primary route of poisoning [14, 18] and diarrhea, vomiting, altered consciousness and epigastric pain are the most common chief complaints of patients as stated by studies included in this review as shown in Table 2.

**Table 2:** Summary of patterns and outcomes of studies included in systematic review

Author/ year	Study setting	Responsible agents	Route of poisoning	No. of cases reviewed	Circumstance (%)			Frequent manifestations at presentation	Treatment (pre-hospital and /or hospital)/further prevention	Reasons for poisoning	outcomes
					I	UI	UD				
Bacha 2015 [17]	Multi-center	Prescribed drugs (29.7%) Hydrocarbon (18.3%) OPP (14.8%) Sodium hypochlorite (12.5%) Alcohol (8.7%)		128	15.5	77.5	7.0	Hypothermia (34.4%) Tachycardia (17.2%) Fever (7.0%)	Atropine (6.3%) for OPP, Gastric lavage (18%), Antacids (53.3%) for detergent poisoning, Milk (83.3%), Induced vomiting (1.6%)		CFR = 0 TAHI = 15.5 h (median)
Teklemariam 2016 [18]	JUSH	HCA (41.7%), OPP (27.2%) Drugs (12.6%)	Oral ingestion (97.1%)	103	50.5 .	27.2	22.3	Diarrhea and vomiting (49.5%), altered consciousness (16.5%)and epigastric pain (13.6%)	GI decontamination (78.6%), Specific antidotes (12.6%), Other managements (8.7%) Psychiatric referral 8.7% and specific education 40.8%	Quarrel (family, marital) (75.9%), psychiatric problem (14.8%) and Substance abuse (9.3%)	LOS = 17.7 days(median) CFR = 5.8% TAHI = 30`-1 h
Adinew 2017 [14]	UOG hospital	OPP (38.46%)	Oral ingestion (88.9%) Inhalation 2.2% unknown 8.9%	90	90			Loss of consciousness (22.2%)	Decontamination methods such as gastric lavage and activated charcoal (45.6%) and atropine (36.7%) for OPP	Family (45.8%) andMarital (16.9%) disharmony, Unsuccessful love affairs (7.3%), Domestic violence (pregnant after raped) 4 (4.8%), Mental disorder 7 (8.4), Being RVI 4 (4.8%), Conflicts in work area (4.8%), Financial problem (4.8%)	CFR = 0 LOS = 0.74 (mean) TAHI = 13`-1 day
Adinew 2016 [20]	UOG hospital	OPP (38.2%), sodium hypochlorite (41.63%), drug (6.9%) and CO (6.0%)	Oral ingestion (83.6%), inhalation (6.4%)	233	57.5	23.2	19.3		Supportive therapy (intestinal lavage, activated charcoal) (60%) atropine (24%) for OPP	Quarrel with family (54.2%) followed by love affairs (18.4%)	CFR 0.43% TAHI = 4.2 h (mean) LOS = 11.26 h (mean)
Melaku 2006 [36]	TASH ICU	OPP		3548							168 (4.7%) admissions and CFR due to OPP 44 (3.9%)
Desalew 2011 [15]	TASH	HCA (43.1%), OPP (21.6%) and Phenobarbitone (10.3%)		116	96.5	3.5		Loss of consciousness (46.3%) Vomiting (23.8%) Epigastric pain (22.5%) Shortness of breath (2 2.5%)	Milk, water anddifferent home remedies(23.3%), Psychiatry consultation (17.2%)	Temporary quarrel (57%) and emotionaldisturbance (26%), underlying mentalillness (13.4%)	CFR is 8.6% LOS = 1 day (mean) TAHI = 3 h (median) Death-OPP (5/25)
Chala 2015 [21]	AHMC	OPP (52.1%), HCA (12.7%) and alcohols (10.3%)		292	36.6	35.6	27.8		GI decontamination (55.6%), specific antidote and atropine (37.8%) OPP and high pressure oxygen for CO (1.7%), Psychiatric referral (8.2%)	social conflict, socio-economic burden, alcohol and drug abuse	CFR is 1.37% and all death were due to complications of OP poisoning
Jemal 2016 [19]	Adama Referral hospital	HCA (41.6%), followed by OPP and drugs		226	81.9	5.8	12.3		GI decontamination (64.6%), Specific antidote (19%), unspecified (11.5%), no management (4.9%)		The overall CFR is 7.5%
Mekonnen 2016 [22]	UOG hospital	Snake bite The		27 adult patients		100		Bleeding complications and Disseminated Intravascular Coagulation			CFR was 4/27 (14.8%) TAHI = after 12 h

*JUMS* Jimma university specialized hospital, *LOS* length of hospital stay, *UOG* University of Gondar, *TASH* TikurAnbessa Specialized Hospital, *ICU* intensive care unit, *CFR* case fatality rate, *I* Intentional, *UI* Un-intentional, *UD* undetermined, *TAHI* Time to arrive health institution after poisoning, *HCA* house hold cleansing agent, *CO* carbonmonoxide

### Treatment of poisoning

Pre-hospital treatment at home with milk, water and different home remedies was reported [15]. In health institutions, gastrointestinal decontamination (Gastric lavage and activated charcoal) and specific antidotes (atropine and diphenhydramine for organophophosphate and carbamazepine poisoning, respectively), oxygen (for carbon monoxide poisoning), antibiotics and polyvalent antivenom (for snakebite) were used to treat acute poisoning patients [14, 17–19, 21, 22]. Besides, for future prevention of poisoning, psychiatric consultation and referral to institution which provides psychiatry service was reported in two studies [14, 15].

### Outcomes

One study scored 1/9 on the NOS, one 2/9, 2 scored 3/9, 3 scored 4/9 and 2 scored 5/9 indicating poor quality (Table 3).Time of presentation to health institution after poisoning, length of hospital stay and case fatality rate reported lies in the ranges between 0.2–24 h, 0.5–17.7 days, 0–14.8%, respectively.

**Table 3:** summary of methodlogy assessment using Newcastle-Ottawa scale

Sudy	Selection (4 tars)	Comparability (2 stars)	Outcome (3 stars)	Total score (9 stars)
Adinew 2016 [20]	1 star	0 star	3 stars	4 stars
Teklemariam 2016 [18]	1 star	0 star	2star	3 stars
Bacha 2015 [17]	1 star	0 star	1 star	2 sstars
Desalew 2011 [15]	1 star	1 star	3 stars	5 stars
Adinew 2017 [14]	1 star	1 star	2 stars	4 star
Chala 2015 [21]	1 star	0 star	2 stars	3 stars
Mekonnen 2016 [22]	0 star	0 star	1 star	1 star
Melaku 2006 [36]	1 star	1 star	2 stars	4 stars
Jemal 2016 [19]	2 stars	1 star	2 stars	5 stars

## Discussions

The highest prevalence of poisoning was observed in persons below age 30. This might be because of prevalent suicidal ideation seen in these productive and economically active age groups. They are the most physically, mentally and socially active age groups, which makes them prone to increased levels of stress as the majority of them are under pressure to support themselves and their families. These make them vulnerable to commit risk taking behaviors. Besides, other reasons for committing suicides in these age groups could be alcohol and drug abuse [4, 14, 18, 21]. With regard to gender, 78% of studies included in this review revealed that higher occurrence of acute poisoning in females than males [14, 15, 17–21]. This is due to the existence of social repression of females seen in some culture and late seeking of medical attention. In Ethiopia, the reason why females attempt suicide at a higher rate than males is hypothesized to be due the cultural mal-practices towards them; most young females are followed and controlled closely by their family as compared to males. These make them more likely to hide certain behaviors, such as intimate relationships with the opposite sex to avoid conflict or disapproval. Unfortunately, when these behaviors are found out, it may cause more family and personal conflict leading to a suicide attempt with different poisons or drugs [4, 20]. This might be one of the underlying reasons why intentional poisoning is reported as more common than unintentional poisoning as seen in our current review. Only one study conducted on snake bite poisoning in 27 adults shown male to female ratio of 8:1 [22]. In Ethiopia males engage in actual activities of farming more commonly than females. As a result males are commonly victimized than females.

Occupation of victims of acute poisoning was reported in only two studies included in this review and farmers (18.8%), unemployed individuals (18.2%), Students (54.3%) and daily laborers (13%) were identified as the predominant victims. Others groups were house wives, waitress, government employees, merchant, prisoner [15, 21]. This can be rationalized in terms of occupational attributes, burden and socio-economic status which affected the victims negatively leading into stress and commit risk taking behaviors. Technological changes in the twentieth century led to extensive synthesis of new chemicals with subsequent expansion of the pharmaceutical market. These brought important changes in the use of medications globally. Many researchers reported that, aside from their benefit, pharmaceutical products predominate in accidents resulting from exposure to toxic agents [23–26]. Now a day, more than 83 million chemical substances are available and approximately 4000 new chemicals are introduced in the world every day. This abundance of chemicals made different poisons substances in different areas to remain a great challenge for the medical profession. These substances that can lead to poisoning vary depending on factors such as geographical area and culture. Most common agents used in poisonings include, pesticides, rodenticides, herbicides, pharmaceutical products, household chemicals, foods, alcohols, plants, traditional medicines and illegal street drugs [27–29].

In our current review, it was revealed that organophosphates and household cleansing agents are the predominant agents of acute poisoning. This is not surprising in that organophosphate compounds (OPCs) are widely used in third world countries like Ethiopia to increase the yield of agriculture products to meet the highly increasing demand of the society. This resulted in increased availability of OPCs which in turn led to increased incidence of ingestion, resulting in increasing suicidal and accidental poisoning [30]. These compounds are popular insecticides and pesticides because of their effectiveness and non-persistence in the environment or body owing to their unstable chemical [31]. Even though, OPP is preventable public health problem, suicides with these chemicals remain a major clinical problem in developing countries, accounting for more than 90% of total poison exposures [32, 33]. Other agents stated as cause of acute poisoning in the included studies are; pharmaceuticals, hydrocarbons, alcohols, snake bites and so on [14, 15, 17–21].

Global Burden of Disease Study (GBD) reported that, worldwide unintentional poisoning was responsible for an estimated 180,000 deaths in 2010. This is equivalent with a mortality rate of 2.6 per 100,000 inhabitants, making poisoning a top 50 cause of death. Compared to 1990, 11% reduction in total deaths and a 34% decrease in the mortality rate were observed. In addition, over 8.9 million DALYs were lost due to poisoning in 2010, which is almost 20% less than in 1990. Despite these apparent reductions, in low- and middle-income countries unintentional poisoning has resulted in 91% deaths and DALYs. Poisoning is also responsible for a significant proportion of deliberate injuries, particularly those that are self-inflicted. It is estimated that deliberate use of pesticides accounts for 23% of self-inflicted injuries globally [29]. Intentional self-harm is the major cause of death from poisoning in developing country accounting for 600,000 deaths in 1990 [27]. In line with this report, in our current review, intentional poisoning was identified responsible for majority of acute poisonings [14, 15, 18, 19, 21].

Oral ingestion is identified as the primary route of poisoning [14, 18]. This might be due the ease of administering poisoning agents orally as compared to other routes. Acutely poisoned patients require accurate assessment and prompt therapy. Early identification of the culprit toxin/s through history and physical examination is and ensuring a protected airway, adequate ventilation and hemodynamic stability through ABC-approach is crucial. Supportive and symptomatic care is cornerstone of treatment in acute poisoning. In addition, procedures to increase the elimination of toxins and a short section covering specific toxins and their antidotes are also included [34]. In our current review, pre-hospital treatments at home with milk, water and different home remedies were reported [15]. In health institutions, gastrointestinal decontamination and specific antidotes (atropine and diphenhydramine for organophophosphate and carbamazepine poisoning respectively), oxygen (for carbon monoxide poisoning), antibiotics and polyvalent antivenom (for snakebite) were used to treat acute poisoning patients [14, 17–19, 21, 22]. Besides, for future prevention of poisoning, psychiatric consultation and referral to institution which provides psychiatry service was reported in two studies [14, 15].

Acute pesticide and drug poisoning is potentially fatal and it is a reason for visiting a health care providers in emergency department setting. Patients with acute drug poisoning require relatively simple care and have good short-term outcome. However, some of these patients are at risk of acute morbidity and poor long-term outcome. In most cases, the needed care is symptomatic, the hospital lengths of stays are less than two days and the primary outcome is good [2, 35]. In our current review, the average hospital length of stay was 9.2 days (0.5–17.7 days) and case fatality rate lies in the range between 0 and 14.8%. These might have occurred due to inaccessibility of nearby health institutions and untimely presentation after acute poisoning. Time of presentation to the health institution after poisoning was reported by some of the articles included and found to lie in the range between 0.2–24 h. To our best knowledge, this is the first comprehensive systematic review reported the epidemiology of acute poisoning in the country. Indeed, this review is not without limitations. All the studies included in this review process are observational studies (cross-sectional, retrospective and case series) and therefore, suffering from confounding factors. Since the studies included are observational studies with a single arm, we could not do meta-analysis to determine the effect size. In addition, the total score of all of the studes were below the average (highest 5 stars) indicating that the quality of the studies included was poor to reach on solid evidence. Hence, we suggest that robust cohort study to determine the epidemiology and clinical outcomes of acute poisoning is urgently needed.

## Conclusion

Our current review result shown that the occurrence of acute poisoning was higher in female than male and common in less than 30 years of age. Organophosphates and household cleansing agents are the predominant agents of acute poisoning followed by pharmaceutical products, hydrocarbons, alcohols, snake bites. Prescribed drugs were found as the predominant cause of poisoning in children. Following drugs, hydrocarbon, Organophosphate, Sodium hypochlorite, Alcohol, Carbon monoxide were found to be the leading cause of poisoning in this age group.

In regard to circumstances of acute poisoning, intentional poisoning was identified responsible for the majority of acute poisonings. Psychiatry problems, quarrel (family, marital, social), family problems, emotional disturbance, and substance abuse were identified as the most common reason for acute poisoning. Oral ingestion was identified as the common primary route of acute poisoning and pre-hospital treatment at home with milk, water and different home remedies were reported. Awareness on proper handling of chemicals and prescribed agents should be forwarded to users of these agents. Strict regulations regarding the storage and sale of pesticides and chemicals should be implemented. Patients with suicidal poisoning should undergo psychiatric consultation to reduce the risk of future attempts in the reduction of morbidity and mortality.

## Appendix

Full searching strategies for PubMed.

*((((((patterns[All Fields] OR (“epidemiology”[Subheading] OR “epidemiology”[All Fields] OR “incidence”[All Fields] OR “incidence”[MeSH Terms])) OR (“epidemiology”[Subheading] OR “epidemiology”[All Fields] OR “prevalence”[All Fields] OR “prevalence”[MeSH Terms])) OR (“epidemiology”[Subheading] OR “epidemiology”[All Fields] OR “epidemiology”[MeSH Terms])) AND (“poisoning”[Subheading] OR “poisoning”[All Fields] OR “poisoning”[MeSH Terms])) OR (“toxicity”[Subheading] OR “toxicity”[All Fields])) OR intoxication[All Fields]) AND (“ethiopia”[MeSH Terms] OR “ethiopia”[All Fields])*

*Full searching strategies for Scopus.*

“*Incidence” OR “prevalence” OR “epidemiology” AND “poisoning” OR “toxicity” OR “intoxication” AND “Ethiopia”.*

*Filters: we didn’t use any filter for limiting publication data. It was just from inception to July 2017.*

## Abbreviations

AHMC: Adama hospital Medical college
CFR: Case fatality rate
CO: Carbomonoxide
ED: Emergency department
GBD: Global burdern of disease
HCA: House hold cleansing agent
I: Intentional
ICU: Intensive care unit
JUMS: Jimma university specialized hospital
LOS: Length of hospital stay
OPP: Organophosphate poisoning
PICs: Poison information center
TAHI: Time to arrive health institution after poisoning
TASH: Tikur Ambessa Specialized hospital
UD: Undetermined
UI: Un-intentional
UOG: University of Gondar
WHO: World health organization
